# An auditory “low road” for threat processing in humans sensitive to fast temporal cues

**DOI:** 10.1016/j.isci.2026.117436

**Published:** 2026-09-04

**Authors:** Martina T. Cinca-Tomás, Emmanouela Kosteletou-Kassotaki, Jordi Costa-Faidella, Carles Escera, Judith Domínguez-Borràs

**Affiliations:** 1 Brainlab-Cognitive Neuroscience Research Group, Department of Clinical Psychology and Psychobiology, University of Barcelona, 08035 Barcelona, Spain; 2 Institute of Neurosciences, University of Barcelona, 08035 Barcelona, Spain; 3 Institut de Recerca Sant Joan de Déu (IRSJD), 08950 Esplugues de Llobregat, Spain

**Keywords:** auditory threat, fast temporal cues, amygdala, subcortical “low road”, fear conditioning

## Abstract

Rapid threat processing is a fundamental and evolutionarily conserved function of the brain. In vision, influential models of emotion propose the existence of a fast subcortical pathway for threat processing, connecting the visual thalamus with the amygdala, driven by coarse visual inputs. In audition, whether such a “shortcut” operates in humans has remained unknown. Here, using psychophysiology and neuroimaging, we provide convergent human evidence for an auditory pathway that is sensitive to fast temporal acoustic cues, previously linked to salient alarm signals and coarse auditory processing. Threatening sounds with fast temporal cues elicited rapid neural and autonomic responses at early post-stimulus latencies. Notably, right amygdala responses to these cues covaried with individual differences in strength of a direct auditory thalamo-amygdala pathway. Together, these findings identify a previously uncharacterized “low road” for auditory threat processing in humans, and establish fast temporal acoustic structure as a key feature supporting rapid affective responses.

## Highlights

• High amplitude-modulated (AM) threat sounds evoked rapid neural and pupil responses
• Fiber density of MGB-BLA pathway predicted amygdala response to high AM threats
• A human auditory thalamo-amygdala pathway may process fast temporal threat cues

## Graphical abstract

**Figure:**
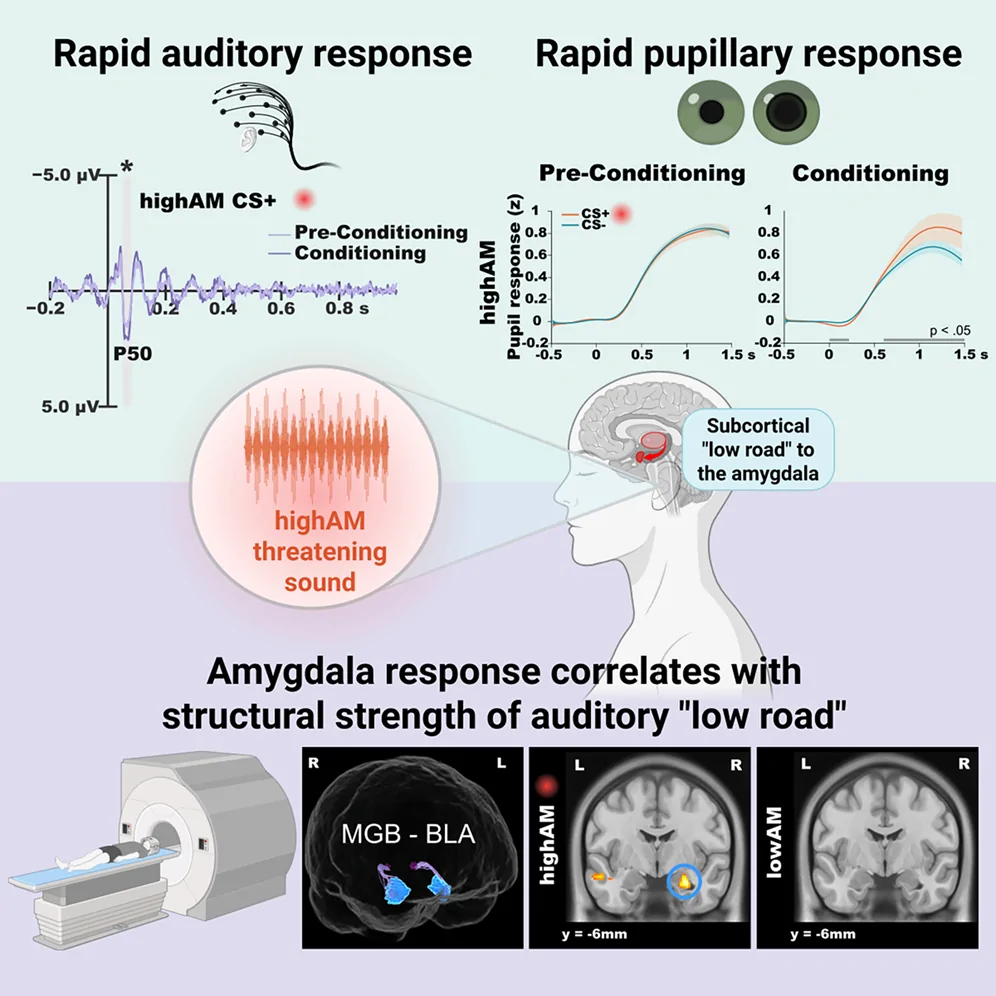

## Teaser

Neuroscience; Systems neuroscience; Sensory neuroscience

## Introduction

Rapid threat processing is one of the most essential and evolutionarily adaptive functions of the human brain. In vision, one of the most extensively studied neural pathways serving this function is the so-called “low road” for emotion processing.^1^^,^^2^ This phylogenetically ancient route is present in most vertebrates^3^ and consists of direct anatomical projections from visual thalamic nuclei to the amygdala (a key structure for detecting danger),^4^^,^^5^^,^^6^^,^^7^ allowing rapid evaluation of threat signals before detailed cortical analysis.^1^^,^^2^^,^^8^^,^^9^^,^^10^ In humans, this neural “shortcut” is thought to rely primarily on coarse visual information (i.e., low-spatial frequencies)^8^^,^^11^^,^^12^^,^^13^ and is functionally associated with magnocellular processing. Consistent with fast information transmission of threat for these type of features, human electrophysiological studies have revealed earlier amygdala and cortical responses when emotional faces contain low-spatial-frequency (LSF) information^13^^,^^14^^,^^15^(see reviews in Tamietto and Gelder^9^ and Vuilleumier^8^). This route is believed to mediate abundant fear-associated physiological processes and behaviors.^2^^,^^8^^,^^10^^,^^16^ Notably, patients with cortical blindness due to bilateral occipital damage retain unconscious fear processing through preserved subcortical routes,^9^^,^^16^^,^^17^ showing amygdala responses predominantly for LSF fearful faces.^18^^,^^19^ Together, broad evidence supports the existence of a fast visual subcortical pathway that prioritizes coarse information to enable rapid threat processing.

An intriguing question that remains unresolved is whether a comparable fast route for threat processing also operates in human audition. In non-human species,^1^^,^^20^^,^^21^^,^^22^^,^^23^ extensive anatomical and functional evidence points to an auditory “low road,”^24^ with direct projections from the medial geniculate body (MGB, the auditory thalamus) to the basolateral amygdala (BLA), a pathway critical for auditory fear.^22^^,^^24^ Although this pathway is well established in animal models, its preservation and functional relevance in the human brain remain undetermined. This pathway has been linked in non-human animals to neural magnocellular populations sensitive to rapid temporal acoustic features,^25^^,^^26^ such as high rates of amplitude modulation.^27^^,^^28^^,^^29^^,^^30^^,^^31^^,^^32^ These neurons share a functional specialization with visual magnocellular neurons for rapidly conveying coarse sensory information.^30^^,^^33^^,^^34^^,^^35^ In humans, magnocellular divisions of the auditory and visual thalamus have been histologically characterized and functionally linked to fast temporal processing in both sensory modalities.^30^^,^^36^^,^^37^^,^^38^ Importantly, rapid temporal acoustic features have also been proposed as salient properties of biologically relevant alarm signals, such as screams, with strong evolutionary significance.^39^ Together, this evidence suggests that fast subcortical threat pathways may prioritize different forms of coarse sensory information across modalities. However, whether such fast auditory signals are associated with subcortical threat processing in the human brain remains unknown.

Here, we tested whether a fast auditory “low road” responsive to fast temporal cues is associated with rapid threat processing in humans. We combined behavioral, physiological, and neuroimaging measures across two complementary studies. During fear conditioning, participants detected human vocalizations modulated at either high or low amplitude modulation rates, which acquired threatening or neutral value through Pavlovian association. Electroencephalography (EEG) and pupillometry captured early neural and autonomic responses. Based on evidence from the visual domain,^13^^,^^14^^,^^15^ we hypothesized that threat-related EEG and pupillary responses would emerge earlier for high, relative to low, amplitude-modulated voices.^28^^,^^30^^,^^40^ In a separate sample of participants, we combined functional magnetic resonance imaging (fMRI) with diffusion-weighted MRI tractography to identify an auditory thalamo-amygdala (MGB-BLA) pathway and assess its functional association with responses to high versus low amplitude-modulated threat cues. We predicted that neural sensitivity to fast temporal threat cues would covary with individual differences in the structural strength of this pathway. This work identifies a previously uncharacterized subcortical route for threat in the human brain, with temporal acoustic structure as a key feature of fast affective processing.

## Results

### Study 1: Early threat responses to fast temporal cues

#### Behavioral responses to threat-associated voices supported an effective fear conditioning

**Figure 1:**
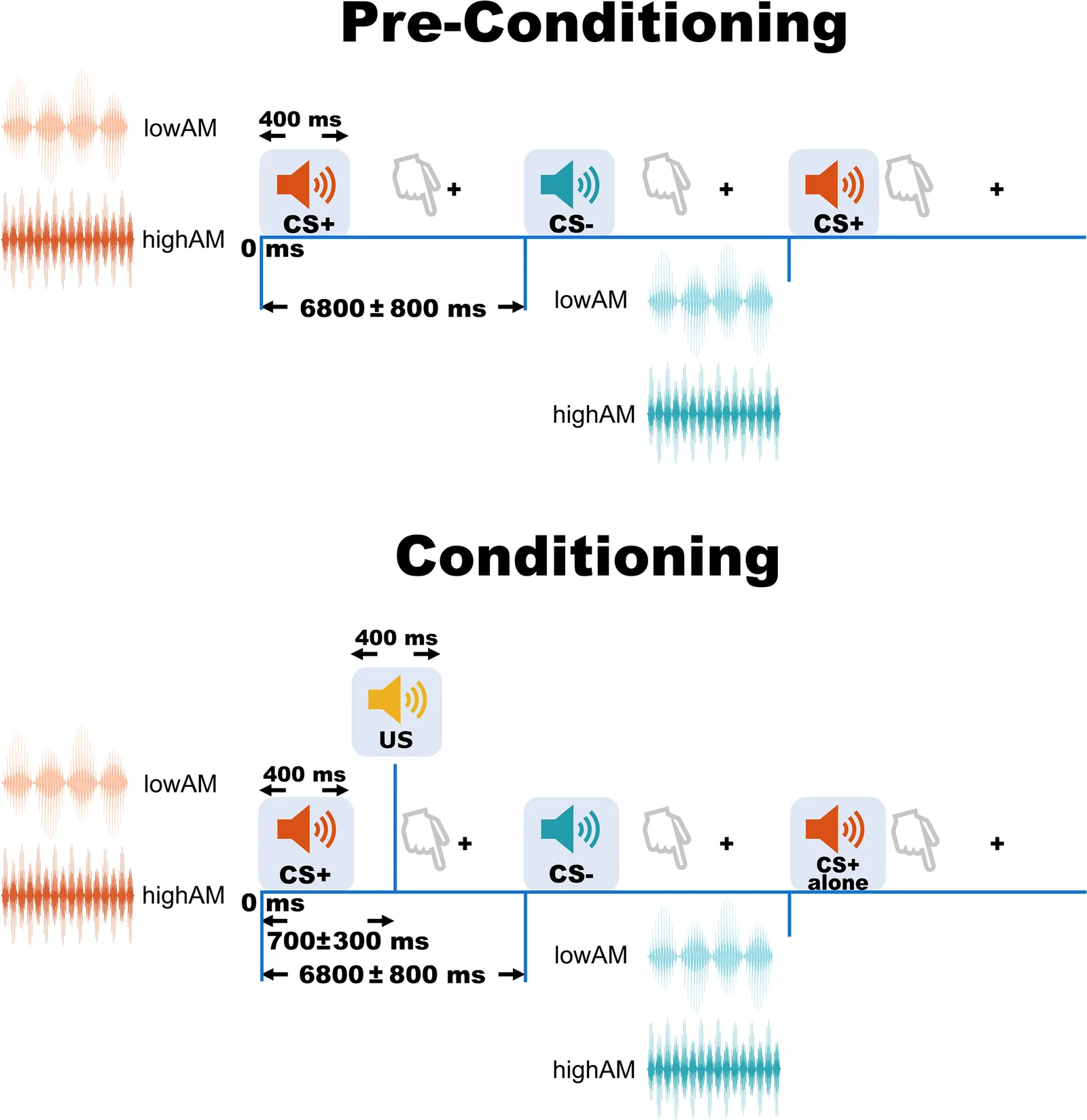
Experimental task During conditioning, CS+ voices were paired with an unpleasant white noise (US), acquiring emotional value. In turn, CS− voices were never followed by the US, remaining neutral. Half of the CS+ trials were presented without the US (CS+ alone), allowing for posterior analysis of the physiological response in the absence of the US. During pre-conditioning, no voices were paired with the US, serving as control stimuli. Participants were asked to indicate whether each voice was presented on the left or right side, while maintaining central fixation. For half of the participants, the CS+ was a female voice, whereas the CS− was a male voice (and the opposite for the remaining sample). All voices were presented at either low or high amplitude modulation rates (lowAM, highAM, respectively).

In the fear conditioning task, participants behaviorally indicated the location of male and female non-verbal voices, both modulated at high (high amplitude modulation [AM]) or low (lowAM) amplitude modulation rates (see STAR Methods). During conditioning, one voice (conditioned stimuli, CS+; a female voice for 50% of participants) was paired with an aversive white noise (unconditioned stimulus [US]), while the other voice type remained unpaired (CS−; a male voice for the same 50% of participants). Before conditioning, the same voices were presented but with no aversive associations, enabling comparisons between physically identical stimuli that differed only in their acquired emotional meaning (Figure 1). Participants learned the association between the CS+ and the occurrence of the US, as revealed by the CS-US contingency scores (main effect of CS: β = 1.960, SE = 0.12, *t*_(81)_ = 15.840, *p* < 0.001, *r*^2^ = 0.64), indicating that they predicted the occurrence of the US more strongly with CS+ stimuli compared to CS− stimuli. There was neither a significant main effect of AM (*p* = 0.182, *r*^2^ = 0.01), nor an AM × CS interaction (*p* = 0.290, *r*^2^ = 0.01). Participants judged emotional (CS+) stimuli as more negative compared to neutral (CS−), as revealed by the valence ratings to voices analyzed on the subtracted values of the conditioning minus the pre-conditioning phase (main effect of CS: β = 0.964, SE = 0.33, *t*_(108)_ = 2.890, *p* < 0.001, *r*^2^ = 0.07). There was neither a significant main effect of AM (*p* = 0.111, *r*^2^ = 0.02), nor a significant AM × CS interaction (*p* = 0.085, *r*^2^ = 0.03). Similarly, participants judged emotional (CS+) stimuli as more alerting than neutral (CS−) stimuli (main effect of CS on subtracted conditioning minus pre-conditioning arousal scores: β = 0.893, SE = 0.24, *t*_(108)_ = 3.641, *p* < 0.001, *r*^2^ = 0.10). However, no significant main effect of AM (*p* = 0.771, *r*^2^ < 0.01) or AM × CS interaction was found (*p* = 0.138, *r*^2^ = 0.02).

Our linear mixed models on the subtracted response time values for the conditioning minus the pre-conditioning phase revealed that participants’ responses were slower for the CS+ compared to the CS− stimuli (main effect of CS: β = 0.067, SE = 0.02, *t*_(81)_ = 3.173, *p* < 0.01, *r*^2^ = 0.02) with no significant main effect of AM (*p* = 0.866, *r*^2^ = 5.45 × 10^-5^) or AM × CS interaction (*p* = 0.568, *r*^2^ = 0.001). A delayed response time for threat cues may be consistent with increased defensive freezing-like processes commonly observed in fear conditioning.^16^^,^^41^^,^^42^ Similar analyses with the hit rate showed that participants were better at detecting the CS+ stimuli relative to the CS− (main effect of CS: β = 0.905, SE = 0.34, *t*_(54)_ = 2.648, *p* = 0.011, *r*^2^ = 0.03). An AM × CS interaction was also observed (β = −1.015, SE = 0.48, *t*_(54)_ = −2.100, *p* = 0.040, *r*^2^ = 0.02), suggesting that facilitated CS+ detection was stronger for highAM aversive sounds compared to lowAM aversive sounds. No significant main effect of AM (*p* = 0.267, *r*^2^ = 0.01) was observed. Behavioral plots are shown in Figure S1.

#### ERP and pupillary responses were increased for all threat-related voices at late time windows

**Figure 2:**
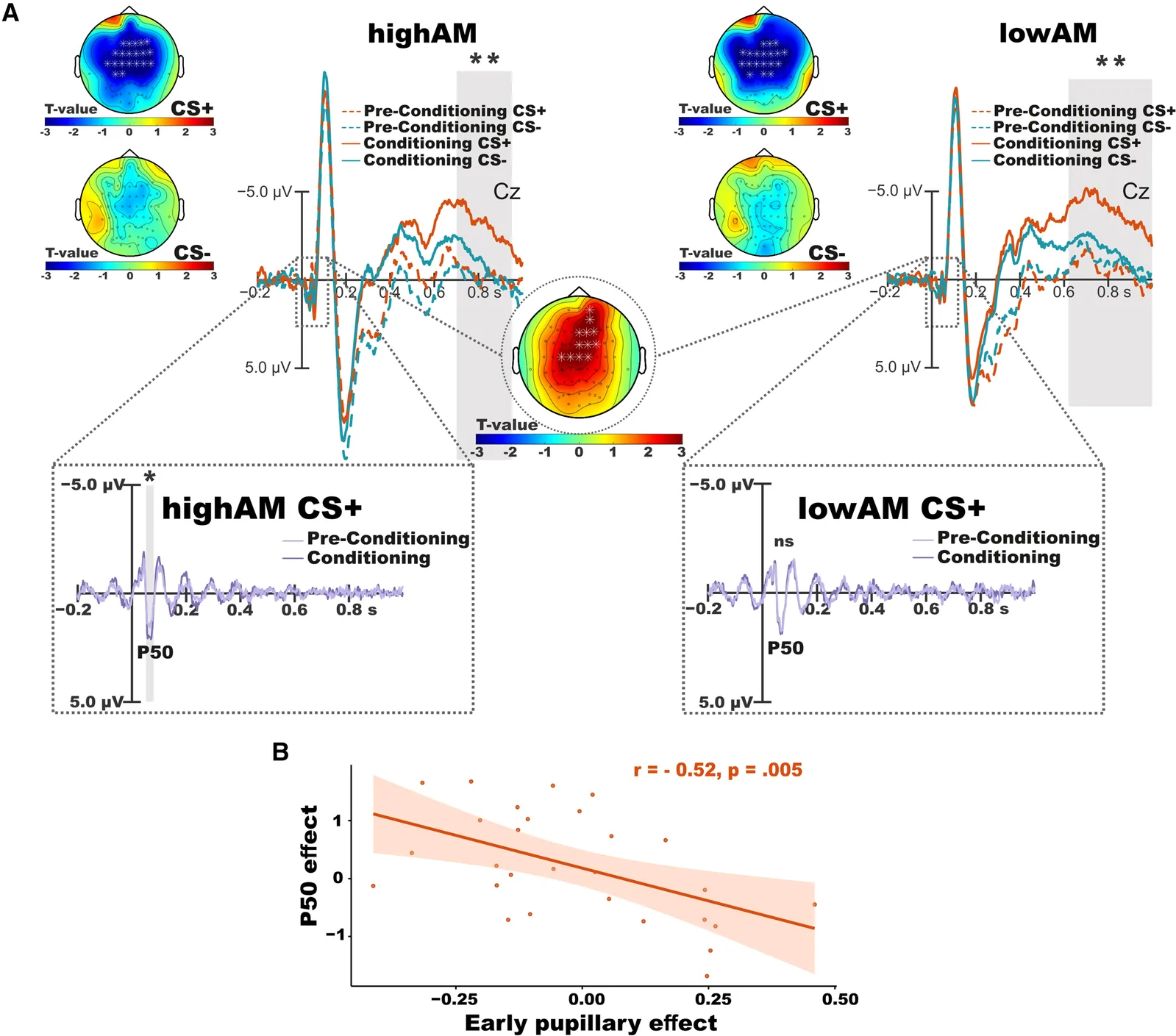
Neural responses to emotional (CS+) versus neutral (CS−) voices (A) Grand-average ERPs for highAM and lowAM voices (*N* = 28). Top: long-latency responses during pre-conditioning and conditioning for both CS+ and CS− voices with topography maps for the significant (708–948 ms) window. Bottom: middle-latency responses (P50) during pre-conditioning and conditioning, and corresponding topography. (B) Association between early pupillary responses (<180 ms) and P50 amplitude (*N* = 28). Shaded areas denote 95% confidence intervals. Gray areas indicate significant effects. ∗*p* ≤ 0.05, ∗∗*p* ≤ 0.01 by cluster-based analysis (A; top) or by linear mixed model (A; bottom). RT, response time; ns, non-significant.

Our cluster-based permutation test on the event-related potentials (ERPs) in response to CS+ versus CS− voices (conditioning minus pre-conditioning phase) revealed a late negative deflection that was larger for CS+ compared to CS−. This effect was maximal over fronto-central electrodes and spread toward parietal areas, and was similar across highAM and lowAM sounds (highAM: 708–948 ms post-stimulus, *t*sum = 12,954.88, *p* = 0.007; lowAM: 622–996 ms post-stimulus, *t*sum = −17,956.20, *p* = 0.007; see Figures 2A and S2). Additionally, brain responses to highAM sounds were generally less negative than lowAM sounds within this late negative deflection over the same electrode regions (main effect of AM: 794–998 ms post-stimulus, *t*sum = 8,272.58, *p* = 0.010). No CS × AM interactions were found within this time window.

**Figure 3:**
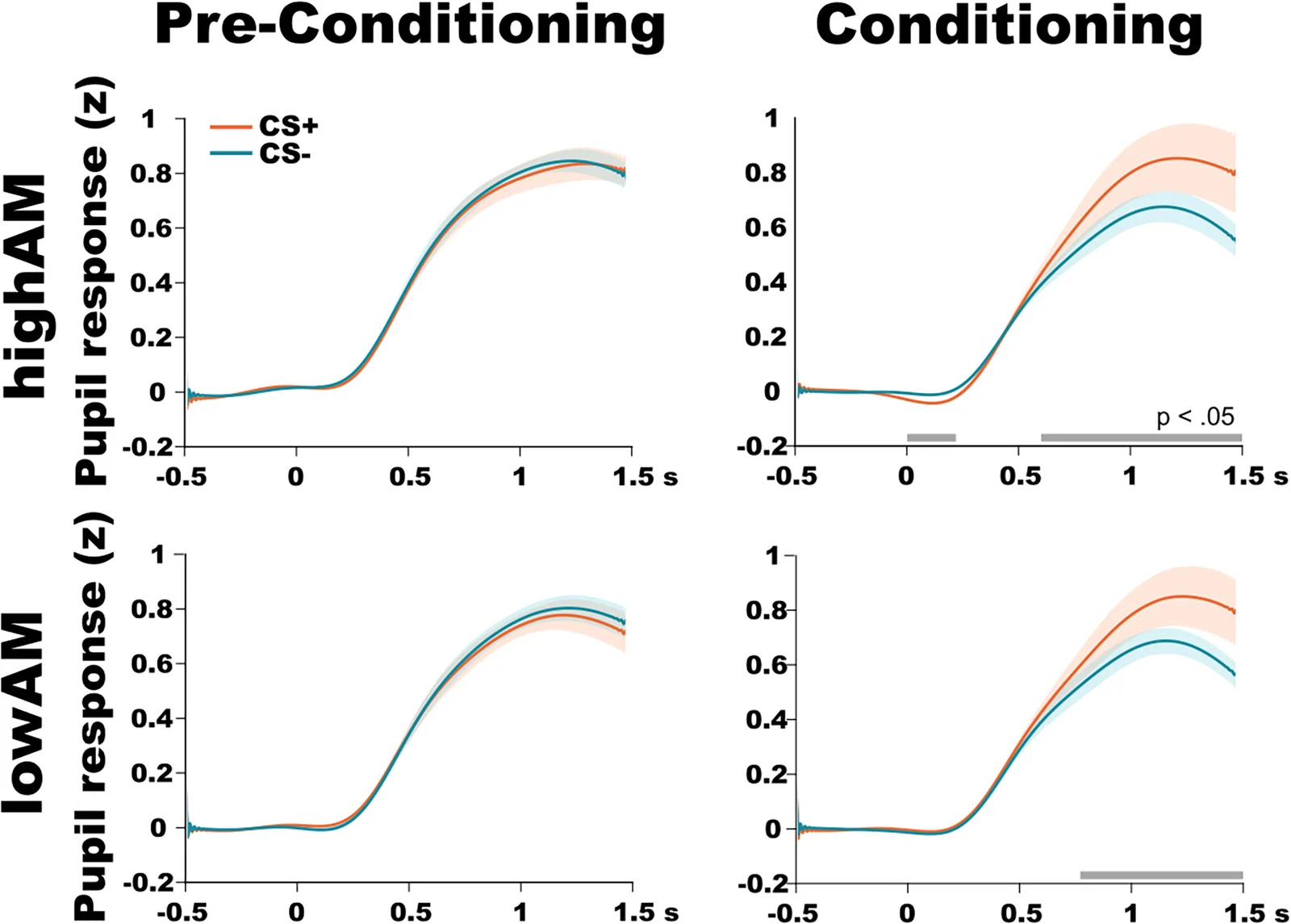
Pupillary responses to the sounds (Top row) Pupil responses to emotional (CS+) and neutral (CS−) voices for each condition and conditioning phase. Data are represented as mean ± SEM. Shaded areas represent SEM. Horizontal grey lines indicate significant effects with *p*-values under 0.05 by cluster-based analysis.

Finally, pupil diameter on the subtracted values (again with subtracted conditioning minus pre-conditioning values) showed a late sustained increase in response to CS+ stimuli, relative to CS–, similarly for both highAM and lowAM stimuli (highAM: 740–1,500 ms post-stimulus, *t*sum = 246.04, *p* = 0.01; lowAM: 790–1,500 ms post-stimulus, *t*sum = 251.27, *p* = 0.01; Figures 3 and S3A), with no statistical difference in this late window among AM conditions (phase × CS × AM: *t*sum = −4.96, *p* = 0 1). A late and sustained increase in ERP and pupillary responses supports an effective attention and arousal response associated with conditioning in participants.

#### Early threat responses were selectively associated with highAM voices

When evaluating conditioning on the long-latency ERPs (Figure 2A), we identified an early phase × CS × AM, significant cluster spanning from 30 to 78 ms over fronto-central electrodes (cluster-level *p* = 0.002, *t*sum = 20,331.10; 10,000 permutations), suggesting that conditioned CS+ sounds elicited an early increase in the auditory response, particularly when presented at highAM, relative to lowAM. For a better approximation of this early effect (as cluster-based statistics may overestimate the timing), we further explored the auditory ERP by applying standardized filters to the EEG signal, so as to isolate the middle-latency responses (Rentzsch et al.^43^; (Figure 2A). The analysis, again implemented directly on the difference wave for conditioning minus pre-conditioning, confirmed a clear amplitude increase of the P50 component that was only present for highAM voices (AM effect: β = −0.230, SE = 0.11, *t*_(82)_ = −2.187, *p* = 0.031, *r*^2^ = 0.03). This increased response to highAM versus lowAM voices occurred irrespective of CS type (CS effect: *p* = 0.99) but emerged selectively during the threat-conditioning phase (conditioning versus pre-conditioning), consistent with a phase-specific threat response.

Remarkably, our cluster-based analysis of the pupillary response also revealed an early reduction in pupil diameter that was specific to the conditioning phase (versus pre-conditioning) and restricted to highAM CS+ voices. This early effect spanned the first 180 ms post-stimulus (CS × AM: 0–180 ms post-stimulus, *p* = 0.024, *t*sum = −45.75; Figures 3 and S3B).

#### No influence of intrinsic physical properties of highAM and lowAM on the observed ERP effects

There were no differences in early neural responses between highAM and lowAM during the pre-conditioning phase, where no emotional (conditioned) stimuli were present. No main or interaction effect was found (main effect of AM: *p* = 0.214, *r*^2^ < 0.01; main effect of CS: *p* = 0.702, *r*^2^ < 0.01; AM × CS: *p* = 0.421, *r*^2^ < 0.01) between the four different conditions during pre-conditioning, suggesting that no effect driven by their intrinsic physical properties was present in the early neural response.

#### Early neural and pupillary responses to highAM threat voices were mutually associated

To further examine the relationship between early neural and autonomic responses to highAM aversive voices, we computed Pearson’s correlation between individual pupil diameter and P50 amplitude values, extracted from the 0 to 220 ms time window, where main effects were observed (each value corresponding to a three-way difference: conditioning minus pre-conditioning, CS+ minus CS− and high AM minus low AM). Pupil responses showed a negative correlation with auditory P50 amplitude (*r* = −0.52, *p* = 0.005, 95% confidence interval [CI]: −0.75, −0.18), indicating that stronger auditory responses to highAM threat voices were associated with greater early pupil constriction (Figure 2B). This suggests a functional association between auditory and pupillary responses to highAM aversive stimuli, potentially reflecting coordinated activity during the initial stages of threat processing.

### Study 2: An MGB-BLA pathway predicts responses to highAM threat cues

#### Amygdala response to highAM versus lowAM threat voices covaried with fiber density of direct MGB-BLA connections

**Figure 4:**
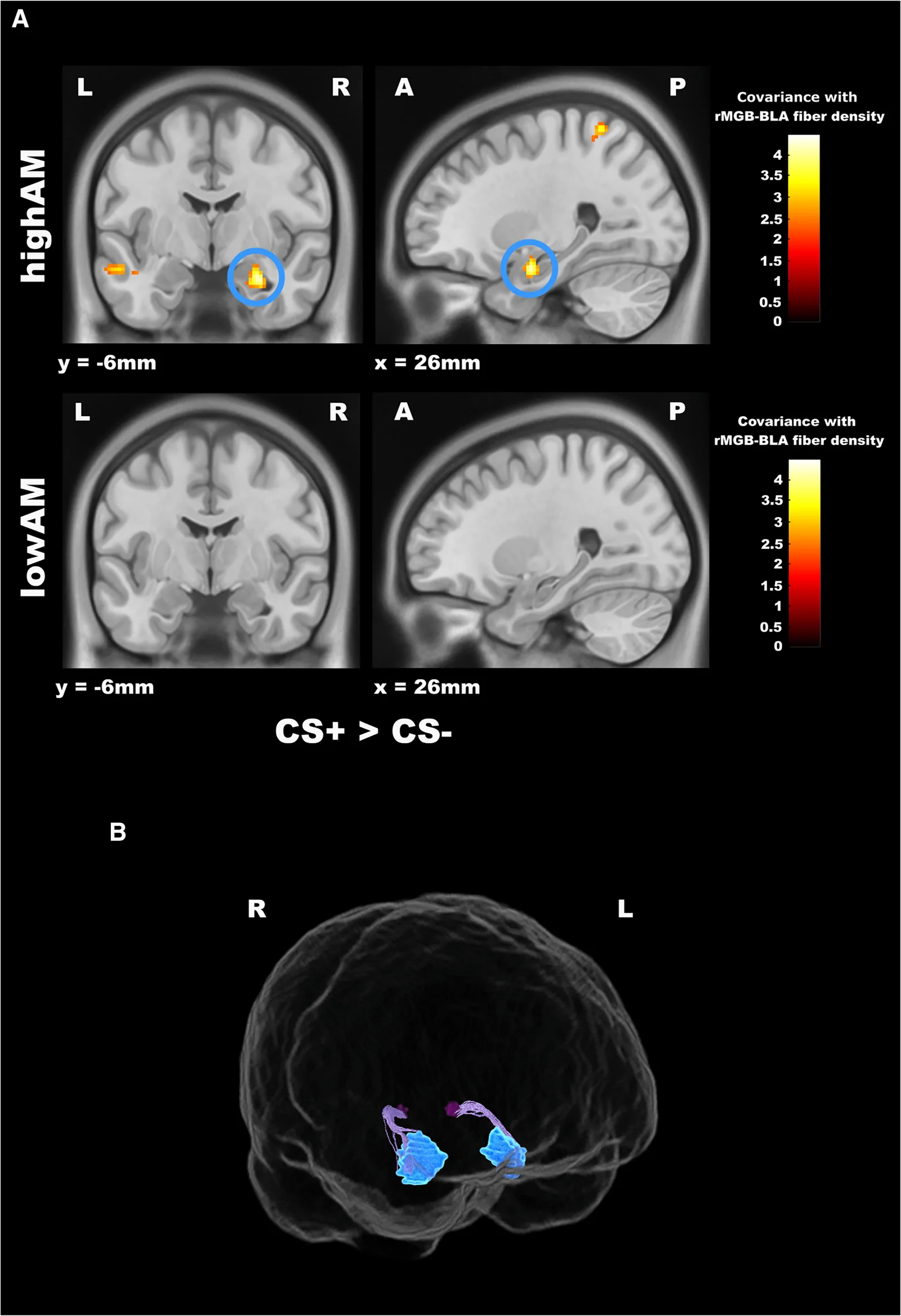
Association between hemodynamic responses to highAM threat and anatomical connectivity of an auditory thalamo-amygdala pathway (A) Functional MRI combined with tractography analysis of connections between the right auditory thalamus (rMGB) and the basolateral amygdala (BLA). Top: right amygdala activity for highAM CS+ > highAM CS− voices that covaried with fiber density of the right MGB-BLA pathway. Bottom: no association for lowAM CS+ > lowAM CS−. L, left; R, right. All contrasts are displayed at *p* < 0.01 and overlaid on the ICBM152 2009b nonlinear asymmetric anatomical template (MNI space) for visualization. The plane coordinates of each slice are indicated bellow each slice. Bright colors represent significance levels of contrasts, as indicated by the scale bars (T values). (B) 3D probabilistic tractography reconstruction of the right and left MGB-BLA pathways in a representative subject. Fibers are seeded from the MGB (dark purple) and terminated in the BLA (blue), with streamlines shown in light purple.

Combining fMRI and diffusion-weighted tractography, we examined whether interindividual variability in the structural strength of direct auditory thalamo-amygdala connections predicted threat-related response to highAM versus lowAM threat cues in the amygdala (see STAR Methods). Analyses were conducted separately by hemisphere to test for potential differences without *a priori* laterality constraints. No association was observed between fiber density of the left MGB-BLA tract and brain responses to threat. In turn, fiber density of the right MGB-BLA tract was not associated with brain activity for the main AM effect of interest (highAM > lowAM). However, fiber density of this right tract covaried with hemodynamic activity in right amygdala for the AM × CS interaction ([highAM CS+ > highAM CS−] – [lowAM CS+ < lowAM CS−]; Montreal Neurological Institute (MNI) coordinates: 28, −6, −22; kE =0 80; T = 4.59; pFWE − SVC < 0.001). In contrast, no such association was observed for the reverse interaction ([highAM CS+ < highAM CS−] – [lowAM CS+ > lowAM CS−]). Post hoc contrasts for highAM emotional cues [highAM CS+ > highAM CS−] confirmed a positive relationship between fiber density of the tract and right amygdala response (MNI coordinates: 26, −6, −20; kE = 0 87; T = 4.23; pFWE − SVC = 0.002; Figure 4; Table 1). This association indicates that individuals with stronger anatomical connectivity between the right MGB and amygdala exhibited greater ipsilateral amygdala response to highAM threat voices. Importantly, this effect was selective to highAM cues, as no comparable association was observed for the lowAM emotional contrast [lowAM CS+ > lowAM CS−] (see Figure 4; Table 1). For completeness, a whole-brain analysis (family wise-error [FWE]-corrected) was also computed for all contrasts of interest, with no significant anatomo-functional associations. Finally, to further address the specificity of the MGB-BLA pathway for highAM versus lowAM threatening sounds, we performed an additional control analysis examining whether similar effects would be observed for the canonical auditory thalamocortical pathway (MGB-primary auditory cortex; PAC; Table 2), used here as a proxy for the cortical “high road.”^1^^,^^2^^,^^44^ In contrast to the MGB-BLA findings, this analysis did not reveal any covariance between the structural strength of the control MGB-PAC pathway and amygdala responses in the critical AM × CS interaction (see Table 1). Such dissociation provides additional support for the specificity of the MGB-BLA pathway for highAM auditory threat, relative to an MGB-PAC cortical pathway. Together, these results provide convergent evidence that a direct auditory thalamo-amygdala pathway is preferentially tuned to highAM threat cues, consistent with engagement of a subcortical auditory pathway.

**Table 1:** Study 2: Effects of rMGB-BLA covariate in amygdala response

Side	Area	*x*	*y*	*z*	T value	k	*p* value
**rMGB-BLA × interaction AM × CS ([highAM CS+ > highAM** C**S−] – [lowAM CS+ < lowAM** C**S−])**							

R	amygdala	28	−6	−22	4.59	80	< 0.001

**rMGB-BLA × reversed interaction AM × CS ([highAM CS+ < highAM CS−] – [lowAM CS+ > lowAM CS−])**							

–	–	–	–	–	–	–	ns

**rMGB-BLA** × **highAM (highAM CS+ > highAM** C**S−)**							

R	amygdala	26	−6	−20	4.23	87	0.002

**rMGB-BLA** × **lowAM (lowAM CS+ > lowAM** CS**−)**							

–	–	–	–	–	–	–	ns

**Control analysis including thalamo-cortical auditory pathway: rMGB-PAC** × **interaction AM** × **CS ([highAM CS+ > highAM** C**S−] – [lowAM CS+ < lowAM** C**S−])**							

–	–	–	–	–	–	–	ns

R, right; ns, non-significant; rMGB, right medial geniculate body; BLA, basolateral amygdala; PAC, primary auditory cortex. All coordinates reported in peak MNI space, and all significant results are reported at *p* < 0.05, corrected for small volume based on amygdala masks from WFU pickatlas (see STAR Methods section).

**Table 2:** Study 2: Tracts and ROIs included to identify them

Tract name	Inclusion ROI 1	Inclusion ROI 2	Exclusion ROIs
MGB − BLA	MGB	BLA	PuA, PuM, PuL, PuI, MD, AV, VA, LD, VPL
MGB − PAC	MGB	PAC	ITG, MTG

MGB, medial geniculate body; BLA, basolateral amygdala; PuA, anterior pulvinar; PuM, medial pulvinar; PuL, lateral pulvinar; PuI, inferior pulvinar; MD, mediodorsal nucleus; AV, anteroventral nucleus; VA, ventral anterior nucleus; LD, laterodorsal nucleus; VPL, ventral posterolateral nucleus; PAC, primary auditory cortex; ITG, inferior temporal gyrus; MTG, middle temporal gyrus; ROI, region of interest.

In sum, threat conditioning modulated behavior, neural, and autonomic responses,^45^^,^^46^^,^^47^^,^^48^ but only highAM voices triggered rapid neural and pupillary effects, compatible with early engagement of the amygdala. Remarkably, the structural strength of a direct right MGB-BLA pathway predicted right amygdala sensitivity to highAM (versus lowAM) threatening voices. These results are compatible with a fast subcortical auditory pathway for threat in humans that may be preferentially engaged by acoustic signals associated with magnocellular processing.

## Discussion

Our findings provide convergent physiological and neuroimaging evidence supporting the involvement of a fast and direct auditory route to the amygdala that is preferentially sensitive to fast temporal (highAM) threat cues. This pathway may thus represent a functional analog of the visual magnocellular “low road” proposed to mediate emotional processing in humans.^2^^,^^9^^,^^49^ Through Pavlovian conditioning, we manipulated both the aversive meaning and the acoustic AM rate of voices, allowing us to isolate physiological responses linked to threat processing for highAM versus lowAM sounds. By extending animal evidence to humans, our results provide a framework for understanding evolutionarily conserved subcortical mechanisms of emotion processing across modalities.

We observed differential behavioral, pupillary, and neural responses both between conditioned (CS+) and non-conditioned (CS−) stimuli, as well as between CS stimuli during conditioning and their identical counterparts before conditioning. Behaviorally, threat-related voices were more strongly associated with the US and were rated as more negative and more arousing than neutral sounds. Participants were also slower but more accurate at detecting them. At the physiological level, late post-stimulus neural and pupillary responses were larger for all threat-related voices. Taken together, these behavioral results, along with the late and sustained increase in neural and pupillary responses, indicate conditioning-dependent enhancements of attention and arousal in participants.^45^^,^^46^

Using EEG and pupillometry, we identified early neural and pupillary responses to threat, and found that these responses depended on highAM, rather than lowAM rates, whereas later responses were comparable across all acoustic conditions. Given that highAM sounds have been associated with magnocellular-related inputs in the auditory system,^28^^,^^30^^,^^32^^,^^37^ our results are compatible with rapid emotion signals that may rely on magnocellular-associated acoustic cues.^8^^,^^11^^,^^12^^,^^13^ This is comparable to fast visual magnocellular-related signals preferentially conveyed by LSF stimuli during emotion processing.^11^^,^^13^^,^^14^ Indeed, magnocellular auditory neurons are considered homologous to magnocellular neurons in the visual system, as both respond to similar parameters, including low-spatial frequencies as well as rapid amplitude and frequency changes over time.^28^^,^^29^^,^^30^^,^^36^ These results may thus be consistent with rapid transmission of affective signals through direct auditory thalamo-amygdala projections previously described in rodents and non-human primates,^50^^,^^51^^,^^52^ which may be sensitive to magnocellular-associated inputs, similarly to vision.

Despite differences in visual and auditory latencies across sensory modalities, our observed increase in amplitude of the auditory P50 component (48–78 ms post-stimulus) for highAM versus lowAM voices is compatible with early human amygdala engagement previously reported in vision for LSF fearful faces,^14^ where rapid amygdala input may transiently enhance cortical excitability for all incoming stimuli before later cortical discrimination.^8^^,^^53^^,^^54^ This is also consistent with amygdala responses preceding those in visual cortex for fearful faces under similar LSF conditions.^13^ More directly, our findings converge with ultrafast responses in the rodent auditory cortex when threatening sounds are processed through the subcortical route to the amygdala.^55^ We note that this P50 effect occurred similarly for CS+ and CS− stimuli, but was specific to the conditioning phase, and therefore associated with threat, possibly reflecting generalized arousal mediated by the amygdala.

In parallel with the neural effects, we observed an early pupillary constriction within the first 180 ms that was also specific to highAM aversive voices. Most prior work on pupil constriction has focused on light-evoked changes or higher level mental processes, such as mental effort.^56^ In the context of the pupillary light reflex, however, evidence in vision shows that threatening subliminal information can enhance the reflex (stronger constriction) under constant luminance,^57^ consistent with rapid, subcortical threat processing without conscious awareness.^8^^,^^16^ Physiologically, the biphasic profile observed here (an early constriction followed by later dilation) is compatible with evidence showing that the BLA projects to the central amygdala, which can in turn influence sympathetic arousal-related responses to threat^4^ via projections to the locus coeruleus, mediating pupil dilation, as well as parasympathetic responses via the periaqueductal gray to the Edinger-Westphal nucleus, mediating pupil constriction.^1^^,^^58^^,^^59^^,^^60^ In line with these ideas, our findings are consistent with a contribution of magnocellular-associated inputs to early autonomic responses, presumably via amygdala circuitry. Accordingly, differential engagement of subcortical circuitry could underlie the rapid pupil dynamics observed for highAM threat, possibly reflecting transient subcortical activity. However, this interpretation remains tentative, and early pupil constriction should not be taken as a definitive marker of subcortical processing. Further research will be needed to disentangle these mechanisms more directly.

Remarkably, a temporal overlap and linear association between enhanced P50 amplitude and early pupil constriction suggested a potential functional link between neural and autonomic responses during the initial stages of threat processing. Thus, participants with stronger P50 amplitude increases to highAM aversive voices also showed greater pupil constriction, suggesting that both responses may originate from coordinated rapid mechanisms supporting adaptive fear behavior. Together, early responses selective to highAM emotional sounds are compatible with rapid threat processing, potentially mediated by fast temporal cues associated with magnocellular processing.^1^^,^^21^^,^^55^ In line with this interpretation, highAM threat cues were also associated with enhanced detection performance, aligning with facilitated detection of threat under these acoustic conditions. However, these associations should again be interpreted with caution given the indirect nature of the measures.

Finally, our multimodal imaging approach allowed us to identify a direct pathway connecting the auditory thalamus (MGB) with the amygdala (BLA) and showed that interindividual variability in the structural strength of this tract covaried with functional amygdala responses to highAM versus lowAM threat voices. This supports a preferential sensitivity of the pathway for fast temporal threat cues that are associated with magnocellular processing, consistent with our physiological findings. In turn, no comparable association was observed for the canonical thalamocortical pathway connecting MGB with PAC, further supporting the specificity of the MGB-BLA pathway for highAM auditory threat.

Moreover, this anatomo-functional association within the MGB-BLA pathway was right-lateralized, consistent with prior visual work implicating the right amygdala in rapid or unconscious threat processing, particularly for low-spatial-frequency stimuli, and thus associated with the visual subcortical “low road.”^17^^,^^18^^,^^61^ In the present context, however, lateralization may depend on the acoustic nature or the temporal dynamics of the stimuli, as well as on their emotional value. In light of these factors, and the lack of consensus on hemispheric specialization for emotion,^62^^,^^63^ we did not formulate a lateralization hypothesis beforehand. Overall, the left hemisphere is typically associated with linguistic processing that conveys word meaning and associated emotional value, whereas the right hemisphere has been linked to paralinguistic information and non-verbal vocal emotions.^64^ Lesion and neuroimaging work further reports amygdala asymmetries, with greater affinity for detailed stimulus-feature extraction on the left and greater involvement in faster, coarser affective analysis of stimuli on the right.^65^^,^^66^ Accordingly, given that hemispheric effects are not uniform across tasks, and that alternative explanations related to task or stimulus features cannot be excluded, the right-sided lateralization observed here should be interpreted with caution. In any case, our thalamo-amygdala connectivity findings converge with animal work demonstrating that direct MGB-BLA projections are involved in auditory fear processing and learning,^20^^,^^22^^,^^25^^,^^26^^,^^51^ and further support their preferential sensitivity to magnocellular-associated acoustic signals.^25^^,^^31^^,^^51^^,^^67^

Although previous work in humans has sought to identify an auditory “low road” using functional or structural connectivity approaches,^44^^,^^68^^,^^69^^,^^70^ this has yet to provide explicit anatomical evidence for the MGB-amygdala association with auditory threat processing. Here, we identify a structurally defined human pathway and characterize its functional association with fast temporal threat cues, advancing current understanding of human subcortical emotion processing.

In human speech, lowAM cues typically convey fine-grained linguistic information, such as syllabic or prosodic cues essential for semantic encoding.^71^ Accordingly, processing of lowAM threat cues may preferentially recruit slower, cortically mediated inputs to the amygdala, analogous to parvocellular channels in vision.^8^^,^^11^^,^^30^^,^^39^^,^^72^ Conversely, highAM cues may preferentially recruit coarse acoustic processing and correlate with sound “roughness,” a hallmark of screams and alarm signals that strongly engage the amygdala.^39^^,^^73^ Thus, a rapid prioritization of highAM threat cues likely reflects their biological salience as warning signals, enabling fast sensory-autonomic coupling^8^^,^^10^^,^^39^ presumably through evolutionarily conserved thalamo-amygdala circuits. Such circuits may be deeply rooted in mammalian evolution, with homologous MGB-amygdala projections supporting rapid defense responses across species.^22^^,^^26^ Importantly, because roughness has been proposed to be an “alarm-like” acoustic feature,^39^ highAM voices could in principle introduce baseline salience effects independent of conditioning.

However, our results do not support a general salience-driven effect by highAM sounds. For instance, the main AM contrast (highAM > lowAM) in Study 2 was not significant, whereas the AM × CS interaction was, indicating that MGB-BLA engagement depended on the acquired threat value rather than highAM stimulation alone. Consistent with this, we did not observe any relationship between MGB-BLA structural connectivity and amygdala responses for these contrasts prior to conditioning. This pattern is thus consistent with a preferential engagement of the pathway for threat processing, rather than for the intrinsic salience of highAM sounds. Nevertheless, while our findings are more consistent with a threat-related engagement of the pathway than with a general salience-driven response, contributions of intrinsic salience associated with roughness may not be fully ruled out.^39^^,^^74^

Importantly, an association of the MGB-BLA tract with highAM cues suggests preferential sensitivity of this tract for amplitude modulations around 40 Hz. However, this does not necessarily imply that thalamo-amygdala connections are never recruited by lower-rate amplitude-modulated cues, or other acoustic parameters or conditions. Indeed, MGB-BLA circuitry in non-human species is known to contribute to numerous threat-related functions.^20^^,^^25^^,^^55^ Similarly, the visual “low road” in humans may be tuned to distinct stimulus features beyond lower or higher spatial frequencies,^75^ including subliminal threat signals,^9^^,^^14^^,^^16^ peripherally presented stimulation,^76^^,^^77^ or even auditory emotional cues.^78^^,^^79^ Future work should disentangle the contribution and specificity of different subcortical routes across affective and sensory domains, clarifying how evolution has shaped threat processing in the human brain.

Finally, the early emotion effects observed for highAM voices may not be explained by physical differences between highAM and lowAM stimuli. Although the faster temporal envelope of highAM sounds could in principle produce earlier peripheral or cortical auditory transduction (see Figure 1), this explanation is unlikely, as no ERP differences emerged between highAM and lowAM voices during pre-conditioning, before emotional associations took place. This rules out trivial acoustic confounds and supports early threat-related modulation.

Importantly, because EEG does not allow localization of amygdala activity, we cannot fully exclude contributions from cortical,^51^^,^^80^ purely thalamic, or brainstem generators^1^^,^^81^^,^^82^ independent of amygdala involvement in our physiological effects. Instead, early amygdala engagement, potentially enabled via a rapid thalamo-amygdala route, may exert a fast modulatory influence, potentially increasing early cortical excitability and contributing to enhanced P50 responses.

Critically, our conclusions do not rely on EEG or pupillometry alone. Instead, our multimodal imaging results provide anatomically specific support for thalamo-amygdala contributions to threat processing under the same acoustic conditions that cannot be obtained from scalp-recorded physiology alone, and align with extensive animal evidence showing a critical role of MGB-BLA projections in affective function.^20^^,^^25^^,^^55^

In sum, our findings provide convergent empirical evidence consistent with the existence of an auditory “low road” in humans that is responsive to magnocellular-associated features, potentially mirroring the subcortical routes long described in vision. This circuitry may reflect an evolutionarily conserved mechanism enabling rapid processing of coarse acoustic threat cues.^39^^,^^73^^,^^83^ Visual and auditory magnocellular systems share response properties and likely derive from common ontogenetic origins,^27^^,^^30^^,^^84^ supporting the idea of ancestral common mechanisms for fast affective processing across modalities. Moreover, given that dysfunction of the visual route and associated processing have been linked to psychiatric conditions such as anxiety, schizophrenia, or autism,^2^^,^^85^ our findings may therefore contribute to understanding early sensory-affective disturbances in the auditory domain.^86^ By revealing a subcortical auditory route sensitive to fast temporal inputs, our work may also bridge animal and human evidence, while contributing to a unified cross-modal framework for emotion processing.

### Limitations of the study

Several limitations of the present study should be noted. First, although our sample size is within the range of standard task-based neuroimaging studies combining fMRI and diffusion MRI, structure-function correlations can be sensitive to sample size and may benefit from replication in larger cohorts. For this reason, the tract-function associations reported here should be interpreted with caution. Importantly, these analyses were hypothesis-driven, restricted to a small number of *a priori* pathways (left and right MGB-BLA), and evaluated using conservative correction procedures, which mitigates concerns related to statistical power and multiple comparisons. Second, the present findings are correlational in nature and do not permit causal inference regarding the directionality of information flow within the MGB-BLA pathway. While the observed associations are consistent with theoretical models of rapid subcortical threat processing, future work using causal or effective connectivity approaches will be necessary to more directly establish functional contributions of this pathway. Third, although participants of both sexes were included, the available sample size did not permit a reliable assessment of potential sex differences. Future studies with larger cohorts will be necessary to determine the generalizability of the present findings across sexes.

## Resource availability

### Lead contact

Requests for further information and resources should be directed to and will be fulfilled by the lead contact, Martina T. Cinca-Tomás (cinca-tomas_t@ub.edu).

### Materials availability

This study did not generate new unique reagents.

### Data and code availability

• Raw data have been deposited at OpenNeuro and are publicly available.
• Study 1: https://doi.org/10.18112/openneuro.ds007114.v1.0.1; Study 2: https://doi.org/10.18112/openneuro.ds007690.v1.0.0.
• Custom MATLAB scripts used for EEG preprocessing, ERP analyses, and cluster-based permutation testing have been deposited at GitHub and are publicly available (https://github.com/MartinaTrisia/SUBEMOcond). Pupillometry analyses were conducted using custom MATLAB scripts implementing the preprocessing procedures described in the STAR Methods. MRI and diffusion MRI analyses were conducted using the standard workflows implemented in SPM12 and MRtrix3, respectively, as detailed in the STAR Methods. Any additional information required to reanalyze the data reported in this paper is available from the lead contact upon request.

## Acknowledgments

This work was funded by the 10.13039/501100000780European Union. Views and opinions expressed are however those of the authors only and do not necessarily reflect those of the European Union or the European Research Council Executive Agency. Neither the 10.13039/501100000780European Union nor the granting authority can be held responsible for them. Supported by ERC grant (HumanSUBthreat, 101088954, to J.D.-B.), the 10.13039/501100004837Spanish Ministry of Science and Innovation project (PID2020-116311GA-I00, to J.D.-B., M.T.C.-T., and E.K.-K.), the María de MaeztuCenter of Excellence (MDM-2017-07-29-20-2, to M.T.C.-T.), Spanish Ministry Predoctoral grant (PRE2021-097083, to E.K.-K.), as well as the 10.13039/501100002809Generalitat de Catalunya (SGR2021-00356, to J.D.-B.).

## Author contributions

M.T.C.-T., conceptualization, investigation, formal analysis, methodology, software, visualization, and writing – original draft; E.K.-K., investigation, formal analysis, software, and writing – review and editing; J.C.-F., technical advice and writing – review and editing; C.E., writing – review and editing; J.D.-B., conceptualization, methodology, supervision, and writing – review and editing. All co-authors have read and approved the final version of the article.

## Declaration of interests

The authors declare no competing interests.

## STAR★Methods

### Key resources table

**Table:** 

REAGENT or RESOURCE	SOURCE	IDENTIFIER
**Deposited data**		

Study 1 data	OpenNeuro	OpenNeuro: https://doi.org/10.18112/openneuro.ds007114.v1.0.1
Study 2 data	OpenNeuro	OpenNeuro: https://doi.org/10.18112/openneuro.ds007690.v1.0.0

**Software and algorithms**		

MATLAB 2019b	MathWorks	RRID:SCR_001622
Psychophysics Toolbox 3	http://psychtoolbox.org/	RRID:SCR_002881
EEGLAB v2022.1	Swartz Center for Computational Neuroscience, UC San Diego	RRID:SCR_007292
Statistical Parametric Mapping (SPM12)	Wellcome Department of Imaging Neuroscience, London, UK	RRID:SCR_007037
R Version 4.2.1	R Foundation	RRID:SCR_000432
FieldTrip	https://www.fieldtriptoolbox.org/	RRID:SCR_004849
FreeSurfer	https://surfer.nmr.mgh.harvard.edu/	RRID:SCR_001847
MRtrix3	https://www.mrtrix.org/	RRID:SCR_024123
original code	this paper	GitHub: https://github.com/MartinaTrisia/SUBEMOcond

### Experimental model and study participant details

#### Study 1: EEG and pupillometry

Thirty-two healthy volunteers were mostly recruited among the University of Barcelona students. All were right-handed, with normal hearing and no previous neurological or psychiatric disorders. Four participants were excluded from the analysis due to a low signal-to-noise ratio on the electrophysiological data. Thus, the final sample consisted of twenty-eight participants (12 females; age range 18-31, mean age = 22 years). All procedures were approved by the ethics committee of the University of Barcelona (RB00003099 - CER042405), in accordance with the Declaration of Helsinki (2024). All volunteers provided written informed consent and received monetary compensation for their participation. Given the small sample sizes, analyses of sex differences were not performed.

#### Study 2: Functional MRI and diffusion tractography

An independent sample of thirty-six healthy volunteers were mostly recruited among the University of Barcelona students. All were right-handed, with normal hearing and no previous neurological or psychiatric disorders. Five participants were excluded from the analysis due to a low signal-to-noise ratio in the MRI data and drowsiness during task performance. Thus, the final sample consisted of thirty-one participants (18 females; age range 18-39, mean age = 24.35). Informed consent was obtained, and all procedures were approved by the ethics committee of the University of Barcelona (RB00003099 *-* CER042405), in accordance with the Declaration of Helsinki (2024). All participants received monetary compensation for their participation. Given the small sample sizes, analyses of sex differences were not performed.

### Method details

#### Study 1: EEG and pupillometry

##### Stimuli

Voices are phylogenetically relevant stimuli and may serve as biologically significant signals^87^^,^^88^^,^^89^ to which the amygdala is especially sensitive.^19^ Two 400-ms excerpts of female and male non-verbal vocal utterances with neutral prosody, extracted from the Montreal Affective Voices database,^90^ served as conditioned stimuli (CS). They were digitally modulated in amplitude at high (40 Hz) and low (10 Hz) amplitude modulation (AM) rates using MATLAB (MathWorks, R2019b) with a 100% AM depth and 10 ms rise and fall ramps, resulting in 4 different stimuli. Voices and AM rates were selected based on previous literature^39^ and preliminary ratings from six independent participants, and those rated as emotionally neutral were chosen. HighAM and lowAM stimuli were designed to differentially engage auditory fast (magnocellular-like) and slow pathways, respectively.^30^ Stimuli were delivered at 80 ± 1 dB SPL on average across participants, while ensuring that they were clearly audible to them. They were presented binaurally and lateralized towards the right or left by manipulating interaural time differences (ITD; Arnal et al.^39^), Additionally, a 400-ms binaural burst of an unpleasant, but not painful, loud white noise served as the unconditioned stimulus (US) individually calibrated (mean = 103 ± 7 dB SPL). All stimuli were delivered through headphones (Sennheiser HD 558) using Psychtoolbox (Version 3.0.14).

##### Procedure and task

The experiment was conducted in a dimly lit room, with participants seated in front of a screen, and their heads stabilized in a chinrest positioned at a distance of 60 cm. Participants were instructed to respond, as quickly and accurately as possible, to which side they heard the voice (left or right, by pressing a keyboard button with their right index or middle finger, respectively), while maintaining their gaze on a central fixation cross. Voices were presented in trials of 6800 ± 800 ms duration (Domínguez-Borràs et al.^41^; see Figure 1). The task followed a classical fear-conditioning paradigm, which depends on sensory inputs to the amygdala.^5^ Conditioning allowed us to confer aversive meaning to acoustic stimuli while independently manipulating their acoustic parameters. Thus, we could compare brain and pupil responses to identical stimuli across conditioning phases (after and before conditioning), as well as across stimulus categories differing in their acquired affective meaning (conditioned/emotional or non-conditioned/neutral), ensuring that emotional effects were dissociated from physical stimulus properties.

The task consisted of a Pre-Conditioning, a Conditioning (Figure 1), and a short Extinction phase (not analyzed) to remove the emotional value of the stimuli at the end of the experiment. During Conditioning, participants were presented with both highAM and lowAM voices, which were either paired (CS+) or unpaired (CS-) with the unpleasant loud white noise (US). Stimulus assignment as either CS+ or CS- was based on voice gender and counterbalanced across participants (i.e., 50% were presented with highAM and lowAM CS+ male voices, and with highAM and lowAM CS- female voices, and vice versa for the other 50%). The CS-US pairing conferred aversive emotional (fear-related) significance to the CS+, whereas the CS- remained neutral.

During Pre-Conditioning, the same CS stimuli were presented without the US, allowing comparisons among identical stimuli before and after they acquired emotional value. To isolate physiological CS+ responses uncontaminated by the US, conditioning was conducted with a 50% partial reinforcement schedule, such that only half of the CS+ trials were presented with the US, resulting in 50% of CS+ stimuli with no US (CS+ alone; see Figure 1).^48^^,^^91^ The final Extinction phase was identical to Pre-Conditioning, but its sole purpose was to allow the stimuli to lose affective value. Thus, there were four conditions of interest (analyzed) in the Conditioning phase (*CS+ alone highAM*, *CS+ alone lowAM*, *CS- highAM*, *CS- lowAM*), and four control conditions in the Pre-Conditioning phase (*CS+ highAM*, *CS+ lowAM*, *CS- highAM*, *CS- lowAM*).

The Pre-Conditioning phase consisted of 208 trials (52 trials per condition, with 26 trials containing left-lateralized voices, and 26 right-lateralized voices), distributed in four blocks. The Conditioning phase comprised 416 trials across nine blocks. This phase included additional CS+ alone trials (52 highAM, 52 lowAM), and CS+ trials followed by the US (52 CS+ highAM, 52 CS+ lowAM). Because the Conditioning phase included more CS+ than CS- trials (due to the addition of CS+ pairings with the US), we increased the number of CS- trials to minimize expectancy or oddball effects arising from this imbalance.^92^ Specifically, the Conditioning phase included 104 CS- highAM and 104 CS- lowAM trials. We further confirmed that any differences in trial numbers across conditions did not bias EEG results by including the number of trials as a covariate (*p* > .05). The additional Extinction phase consisted of 40 trials (5 trials per condition, one block).

Voices were presented in pseudo-random order, ensuring no more than two consecutive repetitions of the same stimulus to prevent habituation.^46^ Voices were presented ensuring that an equal number of voices was perceived on each side. Trials without participants’ responses within 2 s after voice onset were counted as misses. At the end of each block of the Conditioning phase, participants rated on a scale from 1 (never) to 5 (always), how likely each voice of the experiment was to be followed by the US, to assess their awareness of the CS-US contingency. Finally, at the beginning and end of the Conditioning phase, participants rated all voices for valence and arousal on scales from 1 (very positive/low arousal) to 5 (very negative/high arousal).

##### Electrophysiology

EEG activity was recorded at a sampling rate of 500 Hz in a Neuroscan SynAmps RT system (NeuroScan, Compumedics, Charlotte, NC, USA), from 64 Ag/AgCl electrodes placed in a nylon cap following the extended 10-20 system. The horizontal electrooculogram (EOG) was measured with two electrodes placed on the outer canthi of the eyes, and the vertical EOG with two electrodes placed on above and below the left eye, all referenced to the common reference. One electrode placed at the tip of the nose served as online reference and the AFz electrode was used as the ground. All impedances were kept below 10 kΩ.

Preprocessing was done in MATLAB using EEGLab.^93^ Signal was bandpass filtered at 0.1-100 Hz for long latency responses (FIR orders 9056 and 1812, respectively) and at 10-200 Hz for middle latency responses (FIR order 1812), all using a Kaiser window (β=5.653). Based on the 64 electrodes, independent component analysis was used to remove eye movements and muscle activity, using the Second Order Blind Identification (SOBI), after visual inspection. Epochs were defined from − 200 ms to 1000 ms relative to stimulus (CS) onset, and baseline-corrected (200 ms prior to the sound onset). Trials containing the WN (condition CS+) were not included in the analysis. Malfunctioning electrodes were interpolated with spherical interpolation. Epochs exceeding ± 75 μV in more than 60% of the electrodes were discarded. Event-related potentials (ERPs) were obtained by averaging the remaining epochs within each participant for each condition (for trials with correct responses). Average trial counts per condition were 53 ± 9.4 for long-latency responses and 63 ± 3.8 for the middle-latency responses (Table S1).

##### Pupillometry

Pupil diameter was recorded from the left eye using an EyeLink 1000 Desktop Mount (SR Research; sampling rate = 1000Hz) while participants maintained their gaze on a central black fixation cross. The eye tracker was calibrated at the beginning of the experiment and during breaks. Preprocessing and analysis were conducted with custom written MATLAB scripts and Fieldtrip.

Missing data and blinks were detected and linearly interpolated^94^ were padded. Blinks less than 250 ms apart were merged into a single blink. Signal was bandpass filtered (0.05 to 4 Hz third-order Butterworth; Binda et al.^95^) with blinks and saccades removed via linear regression.^94^^,^^96^ Residual pupil time series was z-scored and resampled to 100 Hz. Trials were epoched from −0.5 to +1.5 post-stimulus, baseline corrected (with a 500-ms pre-stimulus) and averaged per condition.

#### Study 2: Functional MRI and diffusion tractography

##### Stimuli

One 400-ms binaural excerpt of a female non-verbal vocal utterance with neutral prosody, extracted from the Montreal Affective Voices database,^90^ served as conditioned stimuli (CS). It was digitally modulated in amplitude at high (40 Hz) and low (10 Hz) AM rates. Stimuli were calibrated for each participant to be well noticeable in the scanner (mean: 20 dB SPL above hearing threshold). Additionally, a 400-ms binaural burst of an unpleasant, but not painful, loud white noise and a pool of different negative images from the International affective picture system (IAPS; Bradley & Lang^97^) served as the US. Images served to reinforce the conditioning effect inside the scanner despite background noise.

##### Procedure and task

Participants were instructed to respond, as quickly and accurately as possible, to which side they heard a voice (left or right, by pressing a keyboard button with their right index or middle finger, respectively), while maintaining their gaze on a permanent fixation cross at the center of the screen. Voices were presented in trials of 5000±800-ms duration.

The task consisted of a fear-conditioning paradigm with a Pre-Conditioning and a Conditioning phase. During Conditioning, participants were presented with both highAM and lowAM voices, and these were either paired (CS+) or unpaired (CS-) with the unpleasant loud white noise and one IAPS aversive image (US). Contrary to Study 1, stimulus assignment as either CS+ or CS- was based on the source location of each voice instead of the gender of the stimulus voice, and similarly counterbalanced across participants (i.e., 50% were presented with highAM and lowAM CS+ left-sided voices, and with highAM and lowAM CS- right-sided voices). The CS-US pairing conferred aversive emotional value to the CS+, whereas the CS- remained neutral. During Pre-Conditioning, the same CS stimuli were presented without the US. The fear conditioning was conducted with a 50% partial reinforcement.

The Pre-Conditioning phase consisted of 104 trials (26 trials per condition), distributed into 3 blocks. The Conditioning phase consisted of 104 trials distributed across 3 blocks. Similarly, as in Study 1, this phase consisted of more trials than in Pre-Conditioning given the presence of additional CS+ alone voices (resulting into 52 CS+ alone highAM, 52 CS+ alone lowAM, but also trials containing the US; 52 CS+ highAM, and 52 CS+ lowAM trials).

Voices were presented in pseudo-random order, ensuring no more than two consecutive repetitions of the same stimulus to prevent habituation.^46^ Voices were delivered binaurally, but with manipulated amplitude differences so they were perceived as lateralized. In turn, if participants did not respond within 2 seconds after voice onset, the response was counted as a miss.

At the end of each block of the Conditioning phase, participants rated, on a scale from 1 (never) to 5 (always), how likely each of the voices used in the experiment was followed by the US, so as to measure their awareness of the CS-US contingency. Finally, at the beginning and at the end of the Conditioning phase, participants were asked to rate all voices for valence and arousal on a scale from 1 (very positive – little stimulating) to 5 (very negative – very stimulating).

##### Data acquisition

Whole-brain MRI data were acquired on a 3T Philips Ingenia CX scanner equipped with a 32-channel whole-head coil, located at the BarcelonaBeta brain research center (https://www.barcelonabeta.org/es), Barcelona, Spain. The imaging protocol included high-resolution structural T1-weighted (T1w) scans, functional MRI (fMRI), and diffusion-weighted imaging (DWI) sequences. The T1w structural images were obtained using a Turbo Field Echo (TFE) sequence with the following parameters: repetition time (TR) = 9.9 ms, echo time (TE) = 4.6 ms, flip angle (FA) = 8°, field of view (FoV) = 240 mm, voxel size = 0.9 mm isotropic, and 200 slices. The functional MRI scans were acquired with TR = 2660 ms, TE = 33 ms, FA = 80°, FoV = 240 mm, voxel size = 1.8 mm isotropic, and 66 slices with a 0.36 mm inter-slice gap. A multiband acceleration factor of 2 was applied to shorten acquisition time. For the diffusion-weighted imaging, a total of 108 volumes were acquired in the anterior-to-posterior phase-encoding direction. This included 96 diffusion-weighted directions distributed across multiple b-values: 8 directions at b = 500 s/mm^2^, 8 at b = 1000 s/mm^2^, 16 at b = 2000 s/mm^2^, and 64 at b = 3000 s/mm^2^. Additionally, 12 non-diffusion-weighted (b = 0 s/mm^2^) images were included in the main acquisition. For distortion and motion correction, two extra b = 0 s/mm^2^ images were acquired with opposite phase-encoding directions (anterior-to-posterior and posterior-to-anterior). DWI acquisition parameters for both the diffusion-weighted and b0 images were as follows: TR = 7463 ms, TE = 89 ms, FA = 90°, voxel size = 1.65 mm isotropic, 81 slices with no gap, and a multiband acceleration factor of 3.

##### Diffusion MRI preprocessing

Preprocessing of diffusion-weighted images was performed using functions from the MRtrix3 software. First, denoising was conducted using the dwidenoise function, which applies patch-based principal component analysis grounded in random matrix theory to exploit data redundancy.^98^^,^^99^ Rician background noise was subsequently removed using mrcalc. Gibbs ringing artifacts were corrected with mrdegibbs.^100^ Susceptibility-induced distortions and motion artifacts were corrected using FSL’s topup and eddy tools,^101^ integrated via the dwifslpreproc function. B1 field inhomogeneity correction was performed using dwibiascorrect, and a brain mask was generated with dwi2mask to constrain subsequent analysis to brain tissue. Finally, the T1w anatomical image was rigidly aligned to the DWI data using ANTs.

We estimated response functions for grey matter, white matter and cerebrospinal fluid (CSF) using the multi-shell multi-tissue constrained spherical deconvolution (MSMT-CSD) algorithm for each participant.^102^ Using these response functions, we then produced individual multi-tissue fiber orientation distribution functions (fODFs) with the MSMT-CSD algorithm, which represent possible fiber directions with corresponding weights in each voxel. Finally, we performed joint bias field correction and global intensity normalization of the multi-tissue compartment parameters using the mtnormalise command in MRtrix3.

##### Region of interest (ROI) definition

ROI definition involved processing each subject's T1w aligned anatomical image to obtain the ROIs for tractography. This process takes as input the subject’s T1w image and predefined ROIs in MNI space, and outputs a segmented T1w image along with the ROIs in individual subject T1w space. Because the T1w image was already rigidly aligned to the DWI data, the resulting ROIs were likewise aligned and suitable for use in tractography analyses.

Cortical and subcortical segmentation of the T1w anatomical images was performed using FreeSurfer (http://surfer.nmr.mgh.harvard.edu/). The medial geniculate body (MGB) ROI was extracted using FreeSurfer’s thalamic segmentation module, which utilizes a probabilistic atlas derived from histological data and high-resolution *ex vivo* MRI.^103^ The basolateral amygdala (BLA) ROI was defined using the amygdala segmentation developed by Saygin et al.^104^ also implemented in FreeSurfer. The lateral, basal, and accessory basal subregions were combined to form a single BLA ROI. To improve the neuroanatomical specificity of tract reconstruction, exclusion ROIs were applied based on the anatomical location and expected connectivity of the MGB-BLA pathway. These exclusion criteria were informed by initial visual inspection and are detailed in Table 2. The primary auditory cortex (PAC) ROI was defined using the Desikan–Killiany atlas labels available in FreeSurfer, based on subject-specific segmentations.

##### Functional MRI preprocessing

We used statistical parametric mapping (SPM) software (version 12; Welcome Department of Cognitive Neurology, London, United Kingdom) for preprocessing and statistical analysis of functional images from our complementary study. Volumes were slice-time–corrected to the first data point and realigned using the “register to mean” option. Anatomical images were coregistered to the functional image by applying a normalized mutual information three-dimensional rigid-body transformation. Brain segmentation and posterior normalization to the Montreal Neurological Institute (MNI) space was performed to the functional images resampled to a 2-mm^3^ voxel size and spatially smoothed with a 6 mm^3^ full width at half-maximum (FWHM) Gaussian kernel.

The interleaved sequence used to acquire functional time series made it a prerequisite to use slice-time correction as a first preprocessing step.^105^ Slice-timing correction methods can successfully compensate for slice-timing effects.^106^

### Quantification and statistical analysis

#### Study 1: EEG and pupillometry

##### Electrophysiology

A mass-univariate nonparametric cluster-based permutation analysis was conducted^107^^,^^108^ with FieldTrip^109^ on ERP amplitudes using a two-dimensional (time x electrode) approach (individual ERP data to compute main effects; individual ERP difference data across conditions to compute the interaction). Clusters were defined via Delaunay triangulation requiring at least two neighboring electrodes. One-tailed dependent t-tests were applied to the amplitudes at each time x electrode point. P-values were estimated by summing t-values of adjacent significant samples (*p* < .05; two-tailed), using 10,000 Monte-Carlo randomizations. Corrected *p*-values below .025 (one-tailed) were considered significant. For each significant cluster, we report its temporal spread, cluster statistic, and *p*-value.

Effects of conditioning, as a measure of the emotional response, were assessed with a CS (CS+ versus CS-) x Phase (Conditioning versus Pre-Conditioning) interaction on difference waves of pairs of conditions. This subtraction-method approach reduced complexity of the statistical models and controlled for physical differences between the CS voices. Thus, we subtracted the CS+ condition in the Conditioning phase versus the same CS+ condition in the Pre-Conditioning phase, and compared this difference to the equivalent subtraction for CS-. Similarly, to test how amplitude modulation (highAM versus lowAM) impacted the emotional response, we computed a Phase x CS x AM interaction with an analogous subtraction approach ([Conditioning CS+ alone highAM minus Pre-Conditioning CS+ highAM] minus [Conditioning CS- highAM minus Pre-Conditioning CS- highAM], and then statistically comparing this double subtraction with the equivalent computation for lowAM). To further explore the early responses observed with this data-driven analysis, we analyzed the middle-latency P50 response (40-80 ms post-stimulus) at electrode Cz.^110^ Phase x CS x AM effects were further assessed with the same subtraction approach using a linear mixed model (LMM) including CS, AM and their interaction as fixed effects, and Participant as a random effect, using the lmer function^111^ of RStudio (R Core Team, 2024). Additionally, to check for possible differences in early neural responses between highAM and lowAM driven by their intrinsic physical properties (see Figure 1), we performed an LMM into the four different sounds during the Pre-Conditioning phase. Each model included CS and AM as fixed effects, with Participant modeled as a random effect. LMMs were chosen for robustness to moderate violations of normality in some of our measures.^112^ All analyses were done after confirming that all the model assumptions were met.

##### Pupillometry

Nonparametric permutation statistics (10,000 Monte-Carlo iterations) were employed as in the EEG analyses, except clusters here were defined along the temporal dimension. Main and interaction effects of CS, Phase, AM were assessed analogously, computing t-values between subtracted conditions and thresholding them at *p* < .05 (two-tailed dependent test). Finally, pupillary responses were correlated with early auditory ERP amplitudes using Pearson’s correlation (two-tailed). All the model assumptions were verified to be met.

##### Behavior

Response times for correct trials (errors and omissions were below 2%) were computed per subject and condition (Figure S1D). Hit rates were analyzed per condition to assess stimulus detection (Figure S1E). For valence and arousal ratings, we computed difference scores by subtracting Pre-Conditioning from Conditioning ratings. Contingency ratings were averaged across all blocks during Conditioning. Valence, arousal and contingency ratings were analyzed separately for each of the four voice conditions (see Figures S1A–S1C). After confirming that all the model assumptions were met, LMMs were applied to behavioral measures using again CS, AM, and CS x AM) as fixed effects, with Participant as a random effect.

Statistical significance in figures is indicated as follows: ∗*p* ≤ .05, ∗∗*p* ≤ .01 and ∗∗∗*p* ≤ .001. Statistical tests used for each analysis are specified in the corresponding figure legends.

#### Study 2: Functional MRI and diffusion tractography

##### Diffusion MRI analysis

Diffusion MRI tractography was conducted using MRtrix3, leveraging both the preprocessed DWI data and anatomically defined ROIs to reconstruct the MGB-BLA pathway bilaterally. Whole-brain tractograms were first generated using the iFOD2 probabilistic tractography algorithm, seeding 10 million streamlines with a fiber orientation distribution (FOD) amplitude cutoff of 0.06. To reduce the incidence of false-positive and prematurely terminated streamlines, we employed the Anatomically Constrained Tractography (ACT) framework using a five-tissue-type (5TT) segmentation of the T1w image (cortical and subcortical gray matter, white matter, CSF, and pathological tissue; Smith et al.^113^). In the same line, we produced ROI to ROI tractograms for each participant planting a seed point 25,000 times in each voxel of the seeding ROI (MGB) with the following parameters: step size 0.3 mm, maximum fiber length 80 mm, minimum fiber length 5 mm, FOD amplitude threshold of 0.1, and angle threshold of 45 degrees. Resulting tractograms were constrained using inclusion and exclusion ROIs (see Table 2), ensuring biologically plausible reconstructions of the MGB-BLA pathway. To quantify structural connectivity, the targeted tractograms were combined with the whole-brain tractogram, and the SIFT2 algorithm was applied. This method assigns a weight to each streamline to ensure quantitative consistency with the underlying fiber density.^114^ Streamline weights were finally multiplied by the global scaling factor μ, computed from the whole-brain tractogram, to convert them into quantitatively meaningful measures of fiber bundle capacity (FBC; referred to as fiber density) that are comparable across subjects. The number of reconstructed streamlines for each hemisphere are reported in Table S2. For the MGB-PAC pathway, ROI-to-ROI tractograms were reconstructed for each participant by seeding 20,000 streamlines per voxel within the seeding ROI (MGB), using the following parameters: cutoff 0.08, maximum fiber length 60 mm, minimum fiber length 15 mm, FOD amplitude threshold of 0.1, and angle threshold of 25 degrees. Resulting tractograms were constrained using exclusion ROIs (see Table 2), to improve anatomical specificity and ensure biologically plausible reconstructions of the MGB-PAC pathway. Fiber bundle capacity (FBC) was calculated using the same procedure as for the other pathways.

##### Functional MRI analysis

For the individual analysis (first level), trial onsets from the eight conditions of interest (see Table S1) and the two conditions in which the US was presented were modeled in the design matrix as separate regressors. To account for movement-related variance, the first-level GLM also included realignment parameters from each session [x, y, and z translations and pitch, roll, and yaw rotations] and the temporal modulator as a covariate of interest.

After model estimation, contrast images were calculated for each experimental condition (versus baseline). The resulting individual contrast images were then entered into a flexible factorial design at the second level. Fiber density values of the left and right MGB-BLA tract, previously extracted for each participant (see above) were included as two continuous covariates, and modeled with factor-specific interaction terms (fiber density × factor AM, with the levels highAM and lowAM and fiber density x factor CS, with levels CS+ and CS-), again computed as the difference between Conditioning and Pre-Conditioning. No additional tracts were tested or entered into the model. We tested whether the strength of the association between fiber density and the hemodynamic response in the amygdala differed across the AM rate (highAM versus lowAM) and CS type (CS+ versus CS-) with special focus on its interaction ([highAM CS+ > highAM CS-] – [lowAM CS+ < lowAM CS-]). To do so, the model included interaction terms between each fiber density and each experimental factor, and contrasts compared the corresponding interaction regressors. To assess for threat-related responses, we focused particularly on amygdala activity.^6^^,^^25^ To this end, small volume correction (SVC) was applied with the anatomical mask of bilateral or right amygdala extracted from the WFU pickatlas (RRID: SCR_007378) when appropriate. Peak-level significance mask was assessed using family-wise-error (FWE) correction for multiple comparisons, with a statistical threshold of *p* < 0.05 FWE-SVC as the significance criterion. For completeness a whole analysis (FWE-corrected) was also computed for the contrasts of interest.

##### MRI figure visualization

Statistical overlays were visualized in SPM12 on the ICBM152 2009b nonlinear asymmetric anatomical template (mni_icbm152_t1_tal_nlin_asym_09b_hires.nii), available from the McConnell Brain Imaging Centre (https://www.bic.mni.mcgill.ca/ServicesAtlases/ICBM152NLin2009) and originally described by Fonov et al.^115^^,^^116^ The template was used only as a standard anatomical background for visualization in MNI space and was not participant-derived. The colored overlays correspond to the second-level SPM statistical results reported in this study. No manual retouching, cloning, warping, selective enhancement, or alteration of the anatomical background or statistical overlays was performed.

## Supplemental information

Document S1. Figures S1–S3 and Tables S1 and S2

Data S1. Source files used to generate Figure 4
