# Supplementary material for: An auditory “low road” for threat processing in humans sensitive to fast temporal cues
Source: iScience. 2026 Sep 4;29(9):117436. doi: 10.1016/j.isci.2026.117436 (PMC13571650; doi:10.1016/j.isci.2026.117436)
Supplement: Supplementary file 1 [file mmc1.pdf]

iScience, Volume *29*

Supplemental information

An auditory “low road” for threat processing in humans sensitive to fast temporal cues

Martina T. Cinca-Tomás, Emmanouela Kosteletou-Kassotaki, Jordi Costa-Faidella, Carles Escera, and Judith Domínguez-Borràs

SUPPLEMENTAL FIGURES

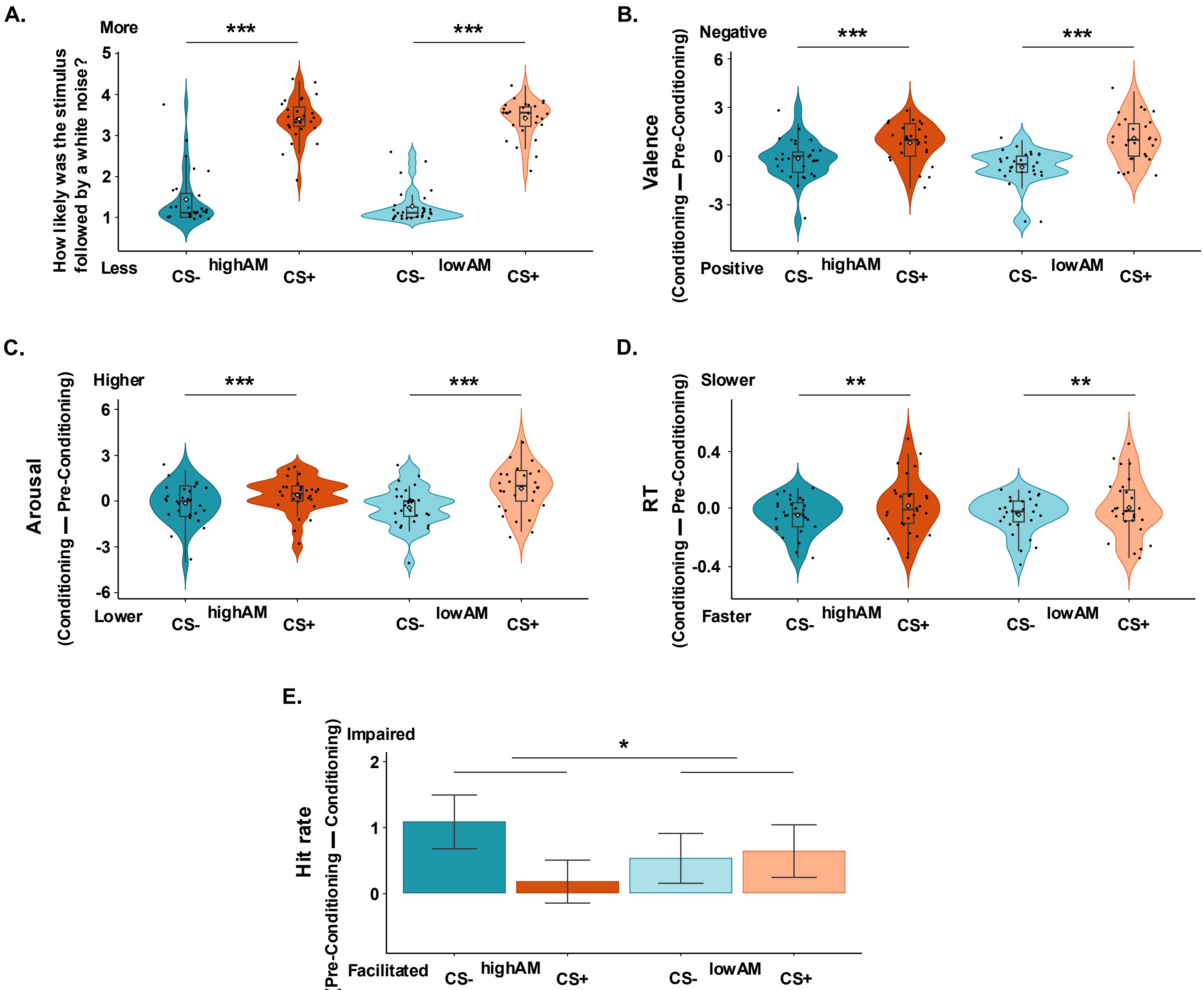

Figure S1. Study 1 behavioral results
Contingency (A), valence (B) and arousal (C) ratings. (D) Response times. (E) Hit rates. (A – D) Violin plots show the distribution of individual participant data. Boxplots indicate the median and interquartile range, and white diamonds indicate the mean. The y axis shows the difference Conditioning – Pre-Conditioning. (E) Bars represent mean ± SEM. The y axis shows the opposite difference (Pre-Conditioning – Conditioning) for a better visualization of the effect. * $p \leq .05$, ** $p \leq .01$, *** $p \leq .001$ by linear mixed model.

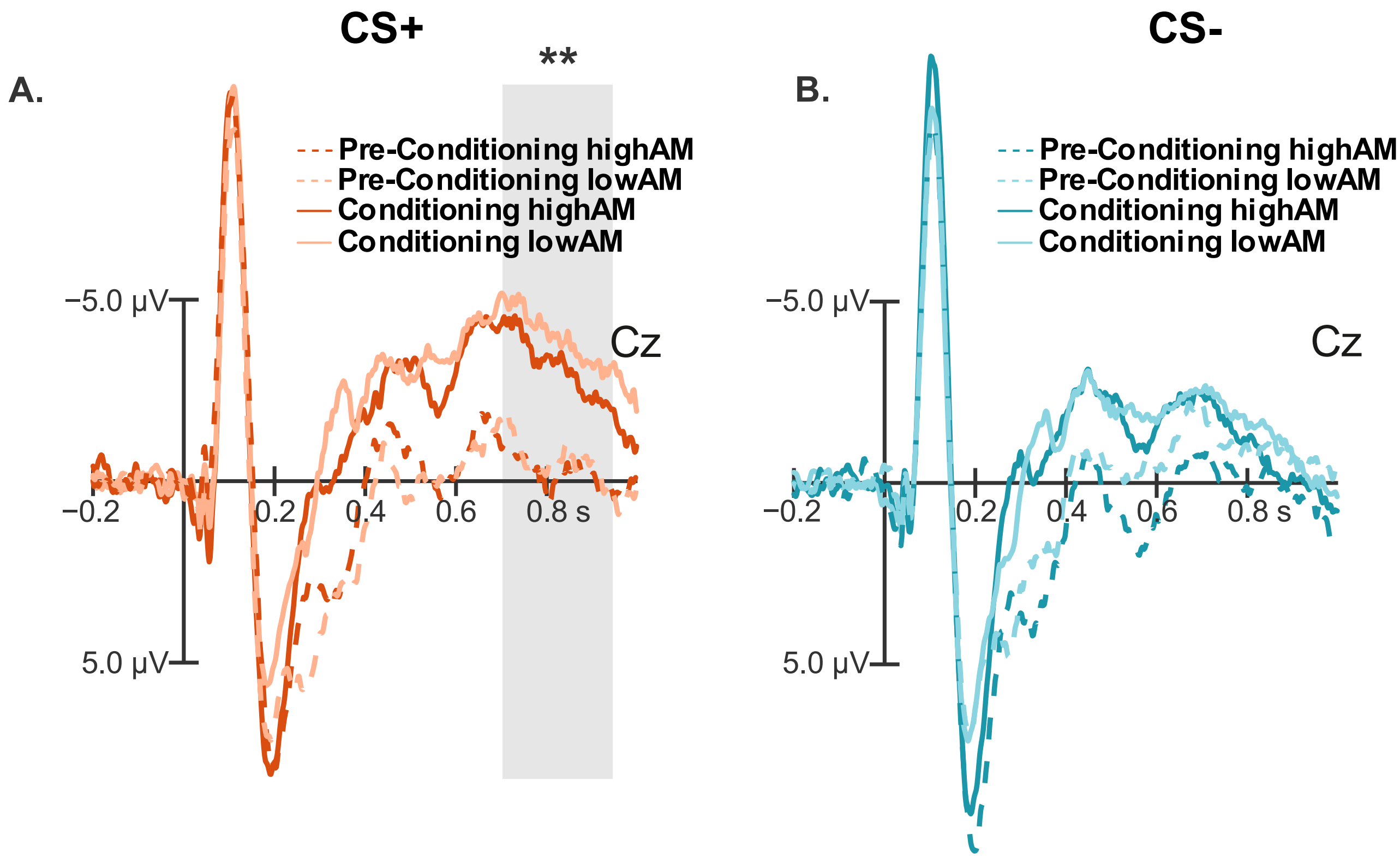

Figure S2. Study 1 electrophysiological data related to Figure 2
(A) Neural responses for emotional (CS+) stimuli. (B) Neural responses for emotional (CS-) stimuli. Grey areas indicate significant effects ** *p* ≤ .01 by cluster-based analysis.

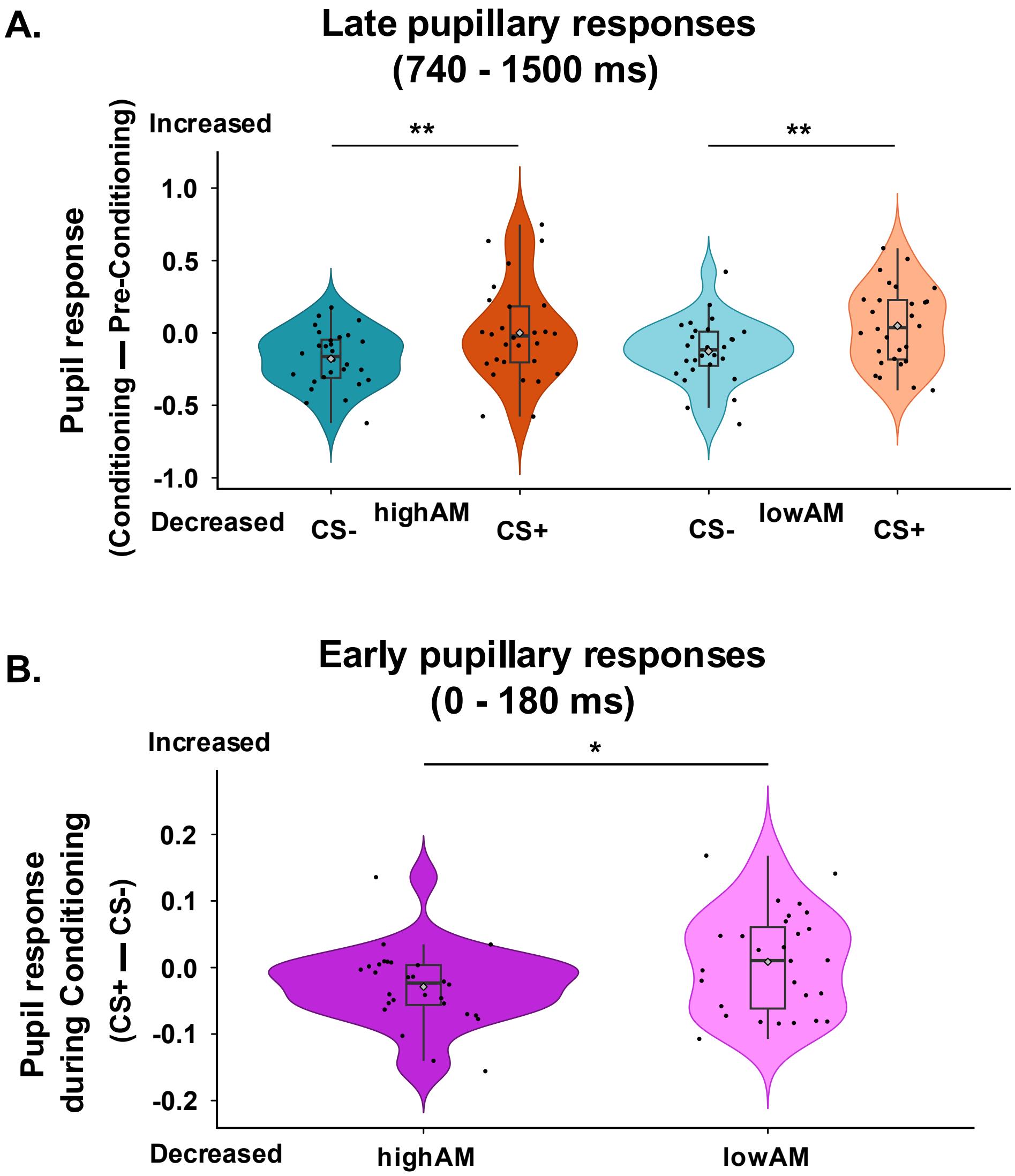

Figure S3. Study 1 pupillary results related to Figure 3
(A) Late pupillary responses (740 - 1500 ms post-stimulus average response). The y axis shows the difference Conditioning – Pre-Conditioning. (B) Early pupillary responses (0 – 180 ms post-stimulus average response). The y axis shows the difference CS+ – CS- during the Conditioning phase. * $p \leq .05$, ** $p \leq .01$ by linear mixed model. In all figures, violin plots show the distribution of individual participant data. Boxplots indicate the median and interquartile range, and white diamonds indicate the mean.

Table S1. Study 1 number of trials accounted per condition for the ERP analyses

	P50 analysis				Full ERP analysis			
Condition	Mean	SD	Min	Max	Mean	SD	Min	Max
highAM CS+ Pre	50.43	2.73	40	52	43.93	6.87	30	52
highAM CS- Pre	50.32	2.58	43	52	43.25	6.61	27	50
lowAM CS+ Pre	50.21	2.79	40	52	43.29	7.03	25	52
lowAM CS- Pre	49.86	2.99	40	52	44.46	5.72	32	52
highAM CS+ Cond	48.93	3.65	38	52	41.61	8.25	23	52
highAM CS- Cond	98.79	5.84	83	104	81.43	16.12	48	104
lowAM CS+ Cond	48.75	3.54	39	52	42.21	8.37	21	52
lowAM CS- Cond	98.32	6.08	82	104	83.46	16.49	47	103

Pre = Pre-Conditioning phase, Cond = Conditioning phase, SD = Standard deviation, Min = Minimum, Max = Maximum, ERP = Event-related potential

Table S2. Study 2 Streamline counts (± SD) per hemisphere for the MGB-BLA pathway

	Number of streamlines	
Tract	Left hemisphere	Right hemisphere
MGB-BLA	408.36 (894.11)	780.03 (2078.94)

MGB = medial geniculate body, BLA = basolateral amygdala. Statistical comparison between hemispheres was not significant.
