# Supplementary material for: An auditory “low road” for threat processing in humans sensitive to fast temporal cues
Source: iScience. 2026 Sep 4;29(9):117436. doi: 10.1016/j.isci.2026.117436 (PMC13571650; doi:10.1016/j.isci.2026.117436)
Supplement: Supplementary file 2 [file mmc2.zip › Data S1/Figure4_lowAM_SPM_Sagittal_export.pdf]

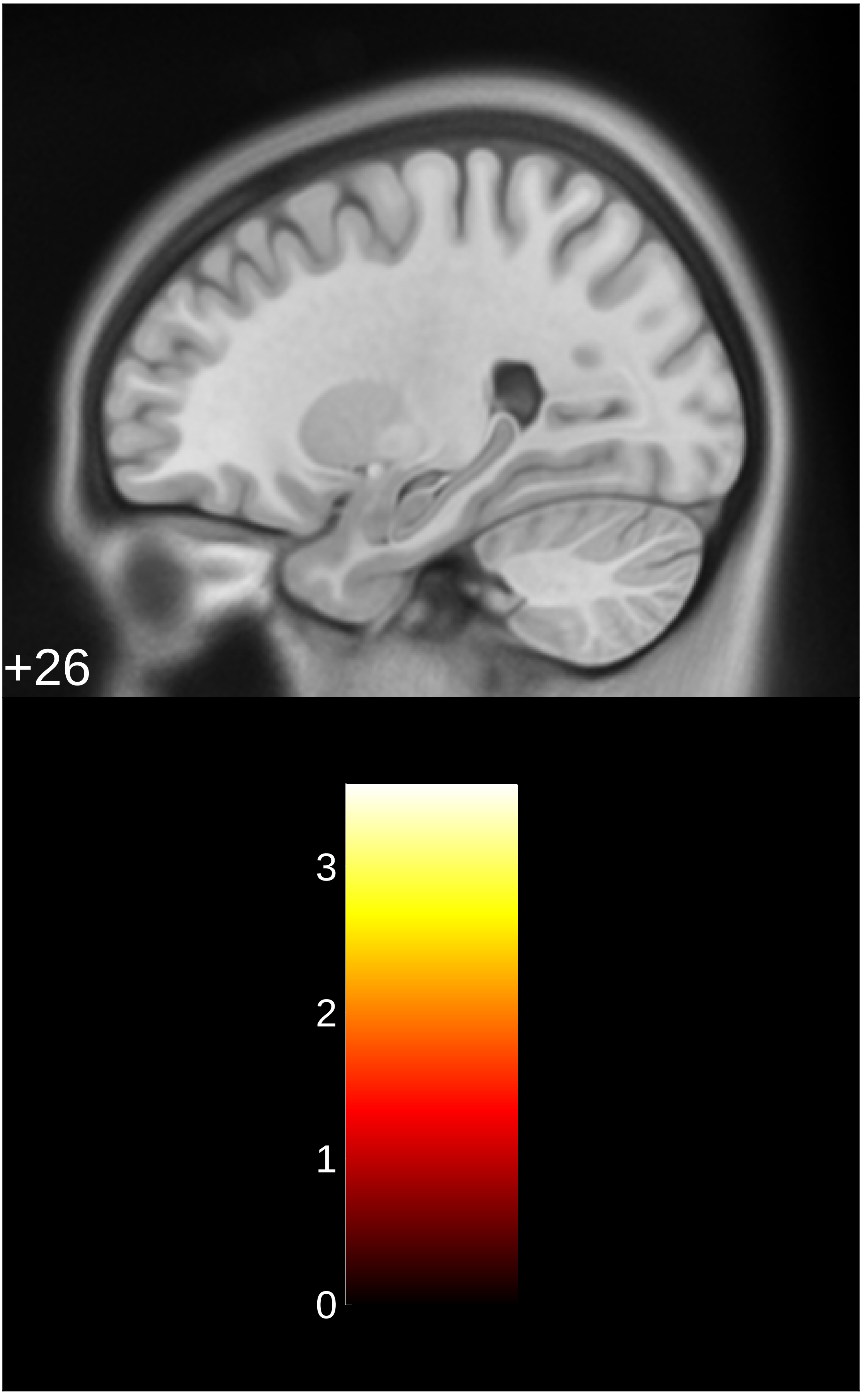
+26
3
2
1
0
